# Editorial: Cardiovascular–kidney–metabolic syndrome: interorgan crosstalk, pathophysiology, and therapeutics

**DOI:** 10.3389/fphys.2026.1845619

**Published:** 2026-05-20

**Authors:** Wei Ding, Hongbao Li, Xiaonan H. Wang, Bin Wang

**Affiliations:** 1 Department of Nephrology, Shanghai Ninth People’s Hospital, School of Medicine, Shanghai Jiaotong University, Shanghai, China; 2 Department of Physiology and Pathophysiology, Xi’an Jiaotong University, Xi’an, China; 3 Department of Medicine, Renal Division, Emory University, Atlanta, GA, United States; 4 Department of Nephrology, Zhongda Hospital, Southeast University, Nanjing, China

**Keywords:** biomarkers, cardiovascular-kidney-metabolic syndrome 1, interorgan crosstalk, pathophysiological mechanisms, risk prediction

Cardiovascular-kidney-metabolic (CKM) syndrome is a clinical syndrome that concurrently involves cardiovascular disease, chronic kidney disease, and metabolic abnormalities. It is characterized by complex pathophysiological mechanisms and represents a major cause of mortality (Li et al., 2025). Recently recognized by the American Heart Association as a distinct clinical entity, CKM syndrome integrates the complex network of pathophysiological processes linking these systems through hemodynamic disturbances, neurohumoral dysregulation, insulin resistance, lipid homeostasis, and inflammatory responses (Marassi and Fadini, 2023; Ndumele et al., 2023). This Research Topic brings together 13 contributions, ranging from fundamental mechanistic studies to clinical risk prediction models, that advance our understanding of CKM syndrome while highlighting the complexity of interorgan crosstalk and the opportunities for therapeutic intervention.

At the most fundamental level, two studies provide critical insights into the cellular processes that underlie CKM syndrome. Zhang et al. demonstrated that PHB2 protects against cisplatin-induced acute kidney injury by preserving mitochondrial integrity, highlighting mitochondrial function as a convergent point in CKM pathophysiology. This finding is particularly significant given that mitochondrial dysfunction has been implicated in insulin resistance, cardiac dysfunction, and renal injury-making it an attractive target for integrated CKM therapies (Narongkiatikhun et al., 2024; Chen et al., 2025). Yuan et al. showed that intermittent fasting mitigates high-fat diet-induced renal injury, with multi-omics analyses implicating thermogenesis, lipid metabolism, inflammation, and insulin resistance in its renoprotective effects. Bonnin-Marquez et al. used kinomic profiling in hyperlipidemic mice with adenine-induced CKD and showed that CKD progression is accompanied by stage-dependent alterations in renal kinase activity, involving pathways related to cell cycle regulation, inflammation, oxidative stress, lipid metabolism, and fibrosis. These mechanistic studies provide biological support for viewing CKM as an integrated disease spectrum and identify potential molecular targets for future therapeutic intervention.

Building on these molecular insights, several studies further elucidated the interorgan communication pathways that drive CKM progression. Zhang et al. showed that hepatic ischemia-reperfusion injury in steatotic livers can lead to secondary acute kidney injury, and that inhibition of hepatic ferroptosis confers indirect renoprotective effects. This liver-kidney axis highlights how injury in one distant organ can propagate through systemic responses to affect others, with ferroptosis emerging as a potential therapeutic target. In addition, Zhu et al. found that intermittent exercise alleviates myocardial infarction-induced renal injury via IGF-1, suggesting that interventions targeting a single organ system may generate cross-organ benefits throughout the CKM network. Together, these bidirectional interactions underscore that CKM syndrome cannot be fully understood by considering individual organ systems in isolation, and they further highlight the need for integrated therapeutic strategies.

It is also worth emphasizing that skeletal muscle may serve as a key regulator within the pathophysiological network of CKM syndrome. Hou et al. reported that myosteatosis is an independent predictor of both all-cause and cardiac mortality in incident dialysis patients. Li et al. showed that lower skeletal muscle density is independently associated with cardiac valve calcification. More notably, Li et al. further found that low skeletal muscle density also predicts constipation in maintenance dialysis patients, suggesting that the consequences of skeletal muscle dysfunction extend beyond traditional cardiovascular endpoints and may affect broader systemic functions. Taken together, these studies further support the view that skeletal muscle is not merely a target organ in the CKM pathophysiological network, but also an important active participant with potential regulatory roles.

At the translational level, two studies improved the accuracy of cardiovascular risk prediction in patients with chronic kidney disease by applying advanced computational approaches. Wang et al. developed an interpretable machine learning model based on body composition parameters to predict cardiovascular mortality in incident dialysis patients, representing an important step toward personalized risk stratification. Similarly, Zhang et al. enhanced the prediction of cardiac dysfunction in hemodialysis patients by integrating bioelectrical impedance analysis (BIA) and physical function assessment. By incorporating objective measures from BIA and physical function assessment, these studies captured important aspects of patient vulnerability that may not be adequately reflected by traditional laboratory markers alone. At the same time, the emphasis on model interpretability addresses one of the key barriers to the clinical implementation of artificial intelligence in medicine.

Several studies also focused on identifying novel biomarkers that may facilitate early detection and risk stratification in CKM syndrome. Fan et al. found that the body roundness index (BRI) independently predicts the risk of incident kidney stones in non-diabetic individuals, highlighting an important link between abnormal fat distribution and renal complications. Zhuang et al. systematically reviewed the association between hypothyroidism and CKD risk, while Guo et al. evaluated the prognostic value of the albumin-corrected anion gap for adverse cardiac events in CKD patients undergoing percutaneous coronary intervention. Taken together, these studies broaden our understanding of CKM-related clinical biomarkers and suggest that future risk assessment should incorporate multidimensional indicators spanning metabolic, endocrine, and electrolyte disturbances.

The 13 articles included in this Research Topic collectively illustrate both the complexity of CKM syndrome and the important progress that has been made in elucidating its pathophysiological mechanisms and advancing its clinical translation. Taken together, these contributions, spanning from fundamental molecular studies to clinical applications, point to several key directions that deserve further attention in future research. First, the foundational importance of mechanistic research cannot be overstated. The studies in this Topic addressing mitochondrial function and metabolic regulation provide a strong biological basis for viewing CKM as an integrated disease spectrum rather than a simple aggregation of coexisting disorders. Second, interorgan crosstalk is both a major challenge in understanding CKM and a potential point of therapeutic breakthrough. Studies of liver-kidney and heart-kidney interactions suggest that organ-specific, single-target approaches may be insufficient to meet the prevention and treatment needs of CKM syndrome, underscoring the need for network-based, system-oriented therapeutic strategies. Third, the recognition of skeletal muscle as a core participant in CKM syndrome opens new avenues for future research and intervention. Exercise-based strategies, nutritional support, and pharmacological therapies aimed at improving muscle quality may confer broad benefits across the entire CKM network. Fourth, the integration of multi-omics data with advanced computational methods provides powerful tools for identifying novel mechanistic pathways and therapeutic targets. The application of metabolomics, transcriptomics, and machine learning reflects a growing shift from mechanistic investigation toward precision translation. Finally, the promotion of clinical validation remains an urgent challenge. Although basic research has proposed several potential interventions, including ferroptosis inhibition and mitochondrial modulation, their clinical efficacy still requires further confirmation. In summary, future progress in the CKM field will require an integrated perspective that considers cardiovascular, renal, and metabolic dysfunction as an interconnected whole, thereby promoting deeper interdisciplinary and integrative research.
